# Everyday Access and Rural Aging in Place: A Mixed-Methods Study of Applied Gerontological Intervention Priorities in South Korea

**DOI:** 10.3390/healthcare14183080

**Published:** 2026-09-19

**Authors:** Kyo-Jeong Chu

**Affiliations:** Department of Social Welfare, Songho University, 210 Namsan-ro, Hoengseong-eup, Hoengseong-gun 25242, Gangwon-do, Republic of Korea; fallkj@songho.ac.kr

**Keywords:** aging in place, rural older adults, everyday access, transportation accessibility, walkability, digital inclusion, applied gerontology, South Korea

## Abstract

**Highlights:**

**What are the main findings?**

- Transportation was the primary everyday access bottleneck, with transfer inconvenience compounded by last-mile walkability and operational safety conditions.
- Focus-group findings contextualized the survey results through repeated transfers, discontinuous access routes, limited non-digital information channels, and local implementation constraints.

**What are the implications of the main findings?**

- Rural intervention planning should combine mobility improvements with last-mile accessibility, wayfinding, assisted access, and parallel digital and non-digital service channels.
- Village-based support requires cross-cutting implementation arrangements, including liability insurance, operating support, professional staffing, and local consultation.

**Abstract:**

**Background/Objectives:** Everyday access to essential services is an important consideration for rural aging in place. This study examined multidomain access barriers and identified applied gerontological intervention priorities in rural South Korea. **Methods:** A quantitatively driven convergent mixed-methods design combined face-to-face survey data from 200 residents across one township center and eight outlying townships with a focus group involving three community welfare council members. Survey analyses described transportation, healthcare, walkability, digital access, safety, and community-care indicators; tested subgroup differences; and used logistic regression to predict high inconvenience with bus service frequency and routes among adults aged 60 years or older (n = 194). Focus-group data were analyzed thematically, and the quantitative and qualitative strands were integrated through narrative comparison and a joint display. **Results:** Transfer convenience was lowest (16.6/100), whereas need for increased service frequency and expanded routes was highest (95.3/100). Healthcare access (29.4/100), walkability (40.9/100), digital access difficulty (67.0/100), and night-time walking safety threats (70.5%) indicated additional barriers. Better walking-environment convenience was associated with lower odds of high transportation inconvenience (adjusted odds ratio = 0.541, 95% CI [0.366, 0.800], *p* = 0.002). Focus-group themes contextualized these patterns through repeated transfers, discontinuous last-mile routes, limited non-digital information channels, and implementation constraints involving insurance, operating support, staffing, and infrastructure. **Conclusions:** The integrated findings suggest that everyday service usability in rural settings reflects interacting transportation, last-mile, information, and operational conditions. Coordinated mobility, walkability, assisted access, digital inclusion, and implementation support may be relevant to aging-in-place planning, although aging-in-place outcomes were not directly measured.

## 1. Introduction

Population aging is reshaping rural communities worldwide, but its implications are particularly acute in dispersed areas where older adults must travel across fragmented service geographies to maintain daily routines, obtain healthcare, and remain socially connected [1,2]. Aging in place has therefore become a central goal of gerontological policy and practice. It refers not simply to staying in one’s home, but to remaining in a familiar environment with sufficient safety, independence, autonomy, and access to needed support [3]. From an applied gerontology perspective, the key question is not whether services formally exist in a rural community, but whether older residents can practically reach, understand, and use them in everyday life.

Rural aging in place is strongly shaped by person–environment fit. In the ecological model of aging, outcomes in later life depend on the alignment between individual capacities and environmental press [4]. This perspective is especially relevant for rural communities because barriers are often cumulative rather than isolated. Transportation limitations, long distances, poor pedestrian routes, limited digital access, and safety concerns can interact to make otherwise available services functionally inaccessible. Recent reviews of rural age-friendly ecosystems similarly emphasize that rural aging-in-place strategies require coordinated attention to transportation, housing, health services, social participation, and community infrastructure [2].

Transportation is a particularly important access pathway for rural older adults. Limited service frequency, insufficient routes, transfer burden, and lack of flexible mobility options can restrict routine trips for healthcare, administration, shopping, and social participation [5,6]. Transportation barriers are also closely linked to healthcare access. Among older adults in rural regions, difficulty reaching healthcare is associated with unmet needs and delayed use of services [7,8]. Flexible route or demand-responsive transportation may reduce some of these barriers, but the practical value of transportation services depends on whether older adults can reach stops, understand routes, and complete the last mile of travel [6,9].

Walkability and last-mile conditions are therefore part of the transportation system, not separate neighborhood amenities. Studies of older adults’ mobility show that sidewalk quality, curb conditions, road crossings, slope, lighting, and perceived safety influence walking for transportation and access to routine destinations [10,11,12,13]. These factors may be magnified in rural areas where pedestrian infrastructure is incomplete and where bus stops, clinics, and administrative facilities may be separated by roads or uneven walking routes. For older adults with mobility limitations, modest last-mile barriers can turn a formally available service into an unusable one.

Digital access has also become a core component of rural aging in place. As health, public, and administrative services become increasingly mediated by websites, mobile applications, online booking systems, and digital information channels, older adults who have limited digital skills or confidence may encounter new barriers to service use [14,15,16,17]. Digital exclusion is not limited to device ownership. It includes interface usability, confidence, accessible information design, and the availability of telephone or in-person alternatives. In rural areas, digital barriers may compound mobility barriers by making it harder to identify routes, check schedules, book services, or complete required procedures.

Universal design and age-friendly community frameworks are useful for addressing these problems, but they must be interpreted broadly in rural settings. Universal design is often associated with physical environments and accessible buildings; however, more recent service and policy perspectives emphasize inclusive systems, accessible information, and intervention areas beyond technical retrofits [18,19,20,21]. For rural aging in place, this means that environmental modification, service connectivity, information access, and operational decisions should be treated as interdependent components of everyday access.

Despite growing evidence on rural aging, several gaps remain. First, many studies examine transportation, walkability, healthcare access, or digital inclusion separately, although older adults encounter these barriers simultaneously in daily life. Second, research often documents needs without translating them into prioritized intervention bundles for local implementation. Third, evidence from rapidly aging East Asian rural communities remains limited in the applied gerontology literature, even though these communities face aging, service centralization, population decline, and digitalization at the same time [22,23,24].

The present study addresses these gaps using data from a dispersed rural county in South Korea. The study aimed to (a) quantify multidomain everyday access barriers related to transportation, healthcare, walkability, digital access, safety, and community care; (b) examine whether these barriers differed by sex, settlement structure, and other individual characteristics; and (c) integrate survey findings with contextual interpretation based on participant accounts to identify applied gerontological intervention priorities for rural aging in place. The study contributes to applied gerontology by reframing rural aging in place as an issue of interdependent everyday access systems rather than single services or isolated facilities, offering an analytically transferable framework for identifying intervention priorities in rapidly aging rural communities beyond the Korean context. In this study, ‘applied gerontological intervention priorities’ are operationally defined as actionable, multi-sectoral community modifications and support mechanisms—spanning transportation, built environments, information channels, and local operational arrangements—that directly target identified systemic bottlenecks to support older adults’ practical functional independence and ability to age in place.

## 2. Materials and Methods

### 2.1. Study Design

This study used a quantitatively driven convergent mixed-methods design to examine everyday access barriers relevant to rural aging in place [25]. Quantitative survey data were used to estimate the magnitude and distribution of barriers across transportation, healthcare access, walkability, digital access, safety, and community care. A focus group with community welfare council members was used to contextualize how these barriers operate in everyday rural life and to identify locally feasible intervention responses. The qualitative component was intended to provide contextual interpretation based on participant accounts rather than to serve as a stand-alone qualitative inquiry seeking thematic saturation. The quantitative and qualitative strands were analyzed separately and integrated at the interpretation stage by comparing survey patterns with focus-group thematic findings concerning local access mechanisms, compounded barriers, and feasible intervention priorities. This integration informed the bottleneck-chain interpretation and the development of intervention bundles for rural aging in place.

The study was based on data originally collected through a county-level needs assessment of universal design and everyday service access. To clearly demarcate the boundary between the primary data collection and the present research: the original county-level assessment collected raw descriptive indicators across broad universal design items solely for local administrative planning purposes. In contrast, the present study represents an independent secondary academic analysis in which I systematically conceptualized and reconstructed these administrative items into multidomain gerontological access frameworks, developed the exploratory Everyday Convenience Barrier Index (ECBI), performed stratified multivariate logistic regression specifically focusing on older adults (aged ≥ 60), and newly executed a convergent mixed-methods synthesis integrating the empirical survey results with focus-group qualitative narratives to identify applied intervention bundles.

### 2.2. Setting and Participants

The study was conducted in Hoengseong-gun, Gangwon Province, South Korea, a rural county with a dispersed settlement structure. The county includes one township center and eight outlying townships. For the quantitative component, face-to-face survey data were collected from 200 community residents across the nine administrative areas in November 2025. Approximately 22 to 23 participants were recruited from each area. The sample was predominantly composed of older adults: 194 of the 200 respondents (97.0%) were aged 60 years or older. All 200 respondents were retained for descriptive and community-level analyses, whereas analyses specifically examining older adults were restricted to respondents aged 60 years or older. For the qualitative component, three township-level community welfare council members were purposively recruited to participate in a focus group. Participants were selected because of their direct knowledge of local service systems, rural living conditions, and the everyday access needs of older residents. Their accounts were used to contextualize the quantitative findings and to identify locally feasible intervention priorities. To protect confidentiality, participants are referred to as Participant 1, Participant 2, and Participant 3. A potential methodological consideration of this sampling approach is that recruiting approximately 22 to 23 participants per administrative division using convenience sampling may not ensure full statistical representativeness across the entire county. In Hoengseong-gun, approximately 20,000 residents are concentrated in the township center (eup), while the remainder of the population and a substantial proportion of older adults are widely dispersed across eight outlying townships (myeon). Although residents in the township center may have relatively better public transit accessibility, the purposive sampling distribution in this study captured all nine administrative areas and appropriately reflected the dispersed rural reality, with 89.0% of respondents residing in outlying townships. Thus, while caution is warranted regarding strict county-wide population-weighted estimates, these findings offer meaningful analytical generalizability to dispersed rural settings where older residents face severe mobility disadvantages.

### 2.3. Focus Group Data Collection

A 90-min, in-person focus group was conducted on 11 November 2025 in a private research office at Songho University. The session was moderated by one researcher. A semi-structured interview guide was used to explore participants’ perceptions of rural transportation, last-mile accessibility, digital and information access, safety, community-based care, and feasible local intervention strategies. With participants’ consent, the discussion was audio-recorded and transcribed verbatim. Identifying information was removed from the transcript prior to analysis, and speakers were labeled Participant 1, Participant 2, and Participant 3.

### 2.4. Ethical Considerations

The data analyzed in this study were originally collected in November 2025 as part of a county-level needs assessment of universal design and everyday service access. The original needs assessment was reviewed by the Institutional Review Board of Songho University and was determined to be exempt from formal IRB review in accordance with institutional requirements, as it was conducted for local service planning and administrative purposes. Participation was voluntary, and informed consent was obtained from all participants prior to data collection. The present study constitutes a secondary research analysis of the previously collected data. The secondary analysis protocol was subsequently reviewed and approved by the Institutional Review Board of Songho University (Approval No. SHU-2026-HR-001). All data used for the present analysis were de-identified.

### 2.5. Research Questions and Hypothesis

The study addressed three research questions. First, how do everyday access barriers relevant to aging in place vary by individual characteristics, including sex, age group, and mobility-aid use? Second, how do these barriers differ between the township center and outlying townships within a dispersed rural settlement structure? Third, when survey findings are integrated with focus-group thematic findings, what applied gerontological intervention priorities emerge for reducing compounded access barriers and supporting rural aging in place?

For the regression analysis, it was hypothesized that greater digital access difficulty would be associated with higher odds of reporting high inconvenience with bus service frequency and routes. Walking-environment convenience was included as an access-related covariate because last-mile conditions may shape the practical usability of public transportation in dispersed rural settings.

### 2.6. Measures and Variables

Most survey items used 5-point Likert-type response scales. Items assessing convenience were coded from 1 = very inconvenient to 5 = very convenient. Items assessing difficulty were aligned so that higher scores indicated greater difficulty when reported as barriers. For descriptive comparability, mean scores on the 5-point Likert-type items were transformed to a 0–100 scale using the formula (x − 1)/4 × 100, where x represents the mean item score. This transformation maps the original scale endpoints of 1 and 5 to 0 and 100, respectively. For convenience items, higher scores indicated greater convenience, whereas for barrier or difficulty items, higher scores indicated greater difficulty or need. Percentages were reported for categorical indicators.

Everyday access domains included physical access to public administrative facilities and essential healthcare facilities; transportation convenience, including transfer convenience and need for increased frequency or expanded routes; walking-environment convenience; night-time walking safety; digital access difficulty; support for village-based community care; and need for barrier-free wayfinding information such as maps and signage.

The primary regression outcome was high inconvenience with bus service frequency and routes. It was coded 1 when respondents selected 1 or 2, corresponding to “very inconvenient” or “inconvenient,” and 0 otherwise. Predictors included sex, age group, settlement structure, mobility-aid use while walking, walking-environment convenience, and digital access difficulty.

### 2.7. Exploratory Everyday Convenience Barrier Index

An exploratory Everyday Convenience Barrier Index (ECBI) was constructed to summarize multidomain access barriers relevant to rural aging in place. The selection of these five specific domains—healthcare access, transportation inconvenience, walking-environment convenience, digital access difficulty, and barrier-free wayfinding—was grounded in preliminary thematic insights from the community welfare practitioners participating in the focus group, who pinpointed these dimensions as the most critical, recurring daily bottlenecks for rural older adults. Furthermore, in accordance with the ecological model of aging, these five indicators capture structural, tangible barriers to functional infrastructure rather than subjective desire or general service needs, thereby delineating practical accessibility deficits from general life-satisfaction ratings. Five indicators were included to represent distinct everyday access bottlenecks: access to essential healthcare, inconvenience with bus service frequency and routes, walking-environment convenience, digital access difficulty, and need for barrier-free wayfinding information. The direction of each component was aligned so that higher values represented greater barriers. Indicators were standardized using z scores and averaged with equal weighting to form the ECBI. The ECBI was intended as an exploratory composite summary of distinct access barriers rather than as a validated psychometric scale or a measure of a single latent construct. Accordingly, internal consistency coefficients were not used to evaluate the index.

### 2.8. Statistical Analysis

Quantitative analyses were conducted using SPSS version 31.0. Descriptive statistics and community-level subgroup analyses were conducted using the full sample of 200 respondents. Group differences in the ECBI were examined using one-way analysis of variance for sex and independent-samples t tests for settlement structure. Effect sizes are reported as eta squared and Cohen’s d. Chi-square tests with Cramer’s V were used for categorical subgroup comparisons, including streetlight operation preference by sex.

Binary logistic regression was used to examine predictors of high inconvenience with bus service frequency and routes. This analysis was restricted to the 194 respondents aged 60 years or older because the study’s regression question specifically concerned older adults and only six respondents were younger than 60 years. Adjusted odds ratios, 95% confidence intervals, and *p* values are reported. Statistical tests were interpreted alongside effect sizes and the applied purpose of identifying intervention priorities rather than solely as hypothesis tests.

### 2.9. Qualitative Contextual Analysis and Integration

**Figure 1:**
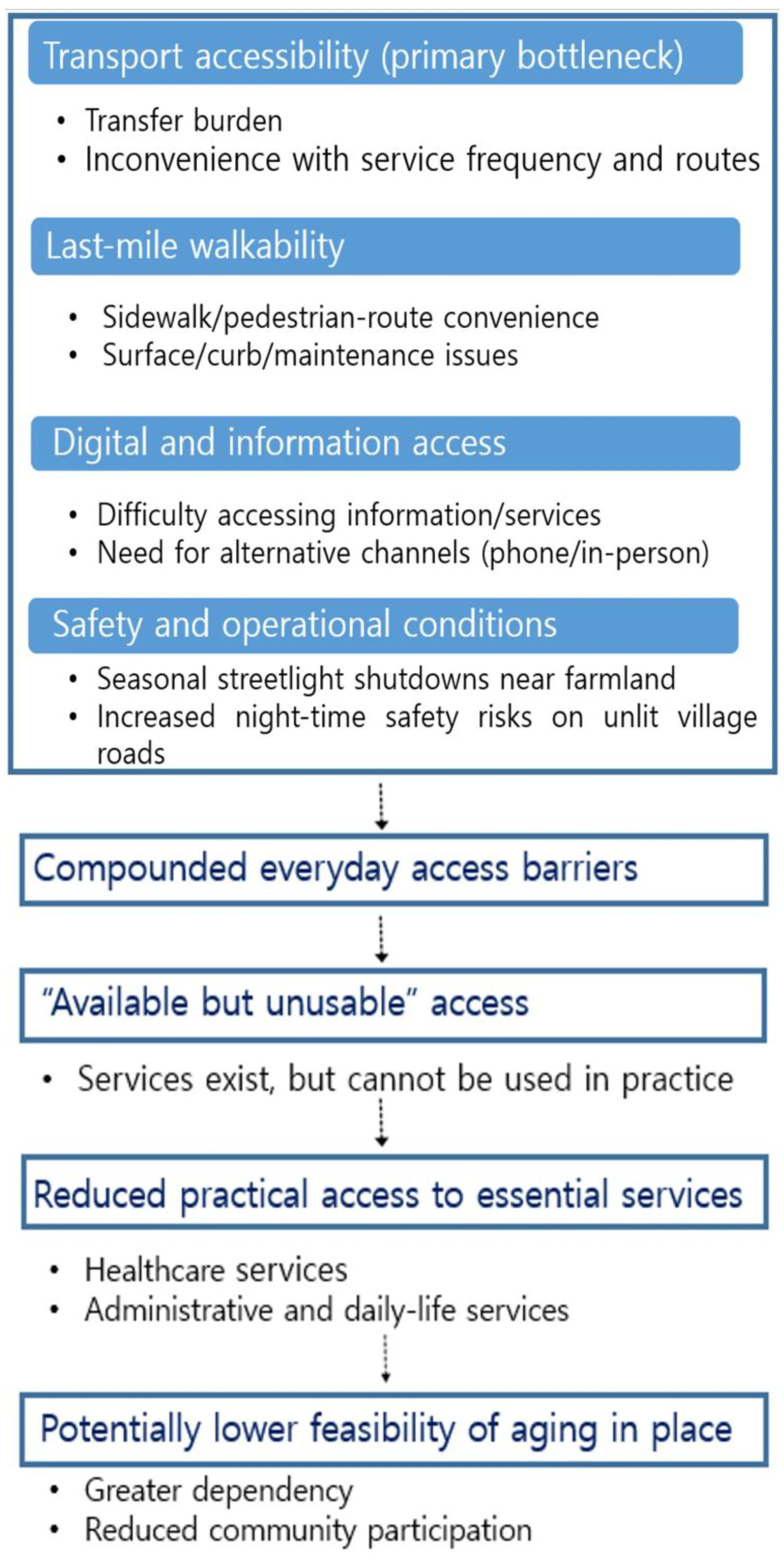
Conceptual bottleneck-chain model of compounded access barriers relevant to aging in place in rural South Korea. The model illustrates how transportation accessibility, last-mile walkability, digital and information access, and safety and operational conditions may jointly contribute to compounded barriers and “available but unusable” access to essential services in dispersed rural settings. These barriers may reduce practical access to healthcare and other everyday services and may, in turn, limit the feasibility of aging in place. Note: Given the cross-sectional nature of the survey, the directional arrows represent a conceptual and heuristic framework synthesizing qualitative insights and empirical associations, rather than empirically verified causal pathways or longitudinal relationships. The final pathway represents a conceptual interpretation because aging-in-place outcomes were not directly measured in this study.

The focus-group transcript was analyzed in Korean using a combined deductive and inductive thematic approach informed by Braun and Clarke [26]. The initial thematic coding was performed by the corresponding author and subsequently triangulated through iterative peer debriefing with a multi-disciplinary research team consisting of two social welfare researchers and one community gerontology practitioner to ensure interpretive rigor and minimize subjective bias. Initial deductive codes were derived from the survey domains and the semi-structured interview guide, including transportation, last-mile accessibility, digital and information access, safety, and community-based care. Inductive coding was then used to identify locally specific mechanisms, interactions among barriers, and feasible intervention responses that were not fully captured by the survey indicators. The transcript was read repeatedly, related codes were grouped into candidate themes, and the themes were reviewed against the full transcript for coherence and distinctiveness. Four themes were finalized: rural transportation constraints and transfer burden; last-mile accessibility and operational safety; digital and information access barriers; and locally embedded and feasible intervention strategies (Figure 1).

The quantitative and qualitative strands were integrated through narrative comparison and a joint display. Survey indicators identified the magnitude and distribution of access barriers, while the focus-group themes contextualized how these barriers operated and became compounded in everyday rural life. Integration was guided by bottleneck-chain logic and was used to identify locally feasible intervention priorities. Illustrative quotations were translated from Korean into English for reporting, with attention to preserving their original meaning.

## 3. Results

### 3.1. Participant Characteristics

Participant characteristics are shown in Table 1. Among the 200 respondents, 64.5% were women and 35.5% were men. The sample was predominantly older: 194 respondents (97.0%) were aged 60 years or older, including 33.0% in their 60s, 43.0% in their 70s, and 21.0% aged 80 years or older. Only six respondents (3.0%) were aged 40 to 59 years. Most respondents lived in outlying townships (89.0%), whereas 11.0% lived in the township center. Mobility-aid use while walking was reported by 34.5% of respondents.

### 3.2. Everyday Access Indicators

Table 2 summarizes key indicators related to everyday access, universal design, and aging in place. Transportation-related indicators showed the clearest bottleneck. Transfer convenience when using local buses was the lowest-rated item at 16.6 on the 0–100 scale, whereas perceived need for increased service frequency and expanded routes was the highest-rated item at 95.3. Access to public administrative facilities was rated relatively favorably at 74.4, but access to essential healthcare facilities was substantially lower at 29.4.

Other domains showed additional barriers. Convenience of sidewalks and pedestrian routes was relatively low at 40.9. Digital access difficulty was high at 67.0, indicating substantial difficulty with digital information or service access. Safety and operational conditions were also salient: 70.5% of respondents reported threats to night-time walking safety. The most frequently preferred streetlight option was wavelength-adjusted lighting designed to minimize crop-growth impacts, selected by 68.0% of respondents. Support for village-based community care for aging in place was high at 85.4, and need for barrier-free wayfinding information was also high at 79.0.

### 3.3. Subgroup Differences

Table 3 presents subgroup comparisons for the ECBI and streetlight operation preference. The ECBI did not differ significantly by sex, F(1, 198) = 2.039, *p* = 0.155, eta squared = 0.010, 95% CI [0.000, 0.054]. The ECBI also did not differ significantly between the township center and outlying townships, t(198) = 0.539, *p* = 0.590, Cohen’s d = 0.122, 95% CI [−0.321, 0.565].

Streetlight operation preferences were similar for men and women, Pearson’s chi-square(3) = 2.323, *p* = 0.508, Cramer’s V = 0.108. Wavelength-adjusted lighting was the most frequently selected option for both groups. These findings suggest that several access barriers and operational preferences were broadly shared rather than concentrated in one sex or settlement subgroup.

### 3.4. Predictors of High Transportation Inconvenience

Table 4 presents the logistic regression model predicting high inconvenience with bus service frequency and routes in the older-adult subsample (n = 194, aged ≥ 60 years). The model was statistically significant, omnibus chi-square(7) = 28.890, *p* < 0.001, with Nagelkerke R squared = 0.188. Better walking-environment convenience was associated with lower odds of reporting high transportation inconvenience, adjusted odds ratio = 0.541, 95% CI [0.366, 0.800], *p* = 0.002. Digital access difficulty was not statistically significantly associated with high transportation inconvenience, adjusted odds ratio = 1.411, 95% CI [0.997, 1.997], *p* = 0.052.

Sex, settlement structure, mobility-aid use, digital access difficulty, and age group were not statistically significant predictors in the adjusted model. Taken together, the regression findings suggest that perceived transportation inconvenience reflects more than bus supply alone. Walking-environment convenience was the only access-related predictor that was statistically significantly associated with transportation inconvenience, whereas the role of digital access remained inconclusive.

### 3.5. Focus-Group Findings

The combined deductive and inductive thematic analysis of the focus-group transcript yielded four themes: rural transportation constraints and transfer burden; last-mile accessibility and operational safety; digital and information access barriers; and locally embedded and feasible intervention strategies. The themes contextualized the survey findings by illustrating how access barriers were understood to operate and become compounded in everyday rural settings and by identifying locally feasible responses proposed by participants.

### 3.6. Rural Transportation Constraints and Transfer Burden

Participants described rural transportation barriers as involving not only infrequent bus services but also route restructuring, repeated transfers, and uneven access to alternative transportation options. Participant 3 explained that bus services in an outlying township had already been limited and were further reduced following a route reorganization. Whereas residents had previously been able to reach the county center without transferring, the revised system required an additional transfer at an intermediate township. This created particular difficulties for older residents who had to repeatedly board and leave buses while carrying groceries or other belongings.

“Bus services were already infrequent, but they were reduced even further after the route reorganization. Previously, residents could travel without transferring, but now older residents have to repeatedly get off and board again while carrying their bags. That is very inconvenient for them.”

(Participant 3)

Participants also described geographic differences in access to alternative transportation. A low-cost taxi program supported essential trips such as shopping and healthcare in some communities, and older residents sometimes shared rides to extend their limited number of eligible trips. However, the service was not available in all outlying townships because its operation depended on the presence of a locally based taxi provider. Consequently, residents of some geographically isolated communities had fewer transportation alternatives despite having substantial mobility needs.

“Our area cannot use the low-cost taxi service because approval requires a taxi operator to be based in the area, and we do not have one.”

(Participant 3)

### 3.7. Last-Mile Accessibility and Operational Safety

Participant accounts indicated that the practical usability of transportation and public spaces depended on the accessibility and safety of the routes connecting homes, bus stops, public facilities, and everyday destinations. Physical barriers included abrupt level changes, steep or curved ramps, obstructed access routes, and surface materials that were difficult to navigate with wheelchairs or strollers. Participant 2 described a ramp at a public office that formally provided accessible access but was difficult to use in practice because of its steep slope, curved design, and vehicles parked near the entrance. Participants also noted that bus stops in more remote villages sometimes consisted only of a roadside sign, without the sheltered facilities available near township centers.

Similar barriers were described in local parks and walking trails. Participant 2 explained that some trails could not be fully used by people using wheelchairs or strollers because the steep approaches and coir-mat surfaces interrupted continuous movement:

“Wheelchair users cannot enter the lakeside trail. The coir mat repeatedly catches the wheels, and strollers face the same problem.”

(Participant 2)

Operational practices also affected night-time walking safety. Participant 2 explained that streetlights located near farmland were often switched off during much of the growing season because residents were concerned that artificial lighting might affect crop production. As a result, older residents returning from bus stops sometimes had to walk long distances along unlit village roads, where encounters with wild animals increased their sense of danger:

“After getting off the bus, some residents have to walk more than one kilometer to reach their homes. During the farming season, the streetlights are turned off, and residents have said that they were terrified when they encountered wild animals beside the road at night.”

(Participant 2)

These accounts illustrated that last-mile barriers arose not only from the absence of accessible infrastructure but also from discontinuities and operational conditions that reduced the usability of existing ramps, bus stops, walking routes, and lighting systems.

### 3.8. Digital and Information Access Barriers

Participants described digital and information barriers as involving both difficulty operating automated systems and limited access to clear and usable service information. Kiosks and unmanned document-issuing machines were viewed as particularly difficult for older residents because their operation involved unfamiliar and complex procedures. Participant 1 emphasized that these difficulties were not limited to people with no experience of digital devices, noting that the systems could be challenging even for other community members:

“Digital exclusion is a problem with kiosks and unmanned terminals. I tried using them myself, and they were genuinely difficult. Operating an unmanned document-issuing machine at a public office is complicated, so I think older adults need some form of training.”

(Participant 1)

Information barriers were also evident in transportation and reservation systems. Participants noted that older residents could not always determine when a bus would arrive or how many times it operated each day because timetable information was not displayed clearly at some stops. Participant 3 suggested that schedules should be presented in large, readable formats so that residents could plan their trips without depending on digital devices or other people. The same participant described similar difficulties with train reservations, which increasingly depended on mobile access and assistance from adult children:

“Older residents cannot make train reservations themselves. Unless their children send the ticket through a mobile phone, the seats may already be sold out. It would reduce their inconvenience if they could make a reservation with a single phone call.”

(Participant 3)

These accounts illustrated that information accessibility depended not only on digital skills but also on whether service information was legible, timely, and available through non-digital or human-assisted channels.

### 3.9. Locally Embedded and Feasible Intervention Strategies

Participants proposed locally embedded interventions that built on existing community organizations, facilities, and routines rather than relying solely on new infrastructure. For rural transportation, they suggested coordinating vehicles operated by local volunteer or community organizations around predictable needs, such as market days and regular hospital visits. Participants emphasized, however, that volunteer willingness alone was insufficient. Sustainable operation required institutional support for fuel expenses, liability insurance, operating schedules, and responsibility in the event of an accident.

Participant 3 further explained that insurance was a major practical barrier to community-provided transportation:

“The biggest problem is insurance because an accident could occur at any time. People may be willing to volunteer, but these issues have to be resolved before they can safely transport residents. Support for fuel expenses would also help.”

(Participant 3)

Participants also identified village halls and senior centers as familiar local platforms for community-based care and service support. Participant 1 described an emerging local initiative in which care could be organized within the village rather than immediately relocating older residents to institutional settings. The participant suggested that existing community facilities could also provide digital assistance and shared activities, although professional staffing would be necessary for sustained care:

“If this approach becomes established, care could be provided through the village hall or senior center rather than sending people directly to a nursing facility. It may allow residents to remain in the place where they have lived.”

(Participant 1)

The discussion also highlighted the need to consider how community spaces were allocated across generations. Participants suggested that village facilities could support both older residents and children, but one participant noted that introducing child-care activities into an existing older-adult space had generated concerns among older residents that their space had been taken away. This indicated that intergenerational use required sufficient space, clear roles, and local consultation rather than simply combining services within one facility.

Additional strategies included publicly identifying accessible restaurants, healthcare facilities, and other local businesses through an accessibility map or information platform. Participants also proposed administrative or financial incentives for new or renovated facilities that reduced physical barriers, along with the inclusion of people with mobility limitations in planning and review processes. These accounts indicated that locally feasible interventions depended on combining community assets with administrative support, clear operating arrangements, accessible information, and the participation of intended users.

### 3.10. Mixed-Methods Integration

Integration of the quantitative and qualitative findings showed convergence across several everyday access domains while also providing complementary explanations of how these barriers operated in the local context. Transportation emerged as the most prominent access bottleneck in the survey, with very low transfer convenience, strong demand for increased service frequency and expanded routes, and low access to essential healthcare facilities. The focus-group findings contextualized these patterns by describing reduced services, repeated transfers, uneven availability of alternative transportation, and the burden of completing essential trips from outlying communities.

The quantitative and qualitative findings also converged in identifying last-mile conditions as an important component of transportation usability. Better walking-environment convenience was associated with lower odds of high transportation inconvenience, while participant accounts described steep or discontinuous access routes, limited bus-stop facilities, and long walking distances between stops and homes. Survey findings concerning night-time walking safety were similarly expanded by accounts of streetlights being switched off because of agricultural concerns and the resulting risks associated with unlit roads and encounters with wild animals.

Digital and information access showed a more complementary pattern. Digital access difficulty was substantial in the descriptive findings, and participants described difficulties with kiosks, automated public-service systems, transportation information, and mobile reservation procedures. However, digital access difficulty was not statistically significantly associated with transportation inconvenience in the adjusted regression model. The integrated findings therefore identify digital and information access as an important independent dimension of everyday service usability, rather than as a confirmed predictor of transportation inconvenience.

Finally, the focus-group findings extended the survey results by identifying locally feasible implementation conditions. Strong support for village-based community care and the need for accessible wayfinding information were complemented by participant proposals involving community-based transportation, village halls and senior centers, telephone and in-person assistance, accessibility information, and institutional support for insurance and operating expenses. Taken together, the integrated findings suggest that everyday access barriers relevant to rural aging-in-place planning may operate as an interconnected access chain and that isolated interventions may be insufficient. Table 5 presents the joint display of quantitative findings, focus-group themes, integrated interpretations, and corresponding intervention priorities.

## 4. Discussion

This study examined multidomain everyday access barriers relevant to rural aging-in-place planning and integrated survey findings with focus-group thematic findings to identify applied gerontological intervention priorities. Across domains, the findings suggest that everyday service usability in dispersed rural settings reflects interdependent access conditions involving transportation, healthcare access, last-mile walkability, digital and information access, wayfinding, and operational safety. The focus-group findings contextualized these quantitative patterns by showing how route restructuring, repeated transfers, discontinuous access routes, limited information channels, and local implementation constraints may make formally available services difficult to use in practice. This integrated pattern supports an access-oriented applied gerontological perspective in which the practical usability of everyday service systems is considered relevant to rural aging-in-place planning.

### 4.1. Everyday Access as a Core Applied Gerontological Issue

The findings suggest that everyday access should be understood as a core issue in applied gerontology. For rural older adults, access barriers are not merely matters of convenience. They may constrain opportunities to maintain daily routines, obtain healthcare, participate in community life, and exercise autonomy in familiar environments. Accordingly, the alignment between older adults’ functional capacities, local service systems, and the everyday environments through which services are reached and used may be one of several conditions relevant to the feasibility of aging in place.

This perspective extends prior aging-in-place research by shifting attention from the presence of services to their practical usability. In dispersed rural communities, a healthcare facility, administrative service, or community program may technically exist but remain difficult to use if transportation is limited, transfers are burdensome, pedestrian routes are unsafe, digital information is inaccessible, or wayfinding support is inadequate. The present findings therefore support an access-oriented approach to rural aging in place, one that considers how service systems, built environments, information pathways, and operational conditions interact in daily life. Recent longitudinal evidence from community-dwelling older adults indicates that accumulated multimorbidity and self-rated health vulnerabilities are inextricably linked to declines in life satisfaction over time [27]. In rural aging contexts, everyday environmental and accessibility barriers may exacerbate these vulnerable health trajectories by isolating older adults from timely healthcare and routine support networks.

### 4.2. Transportation Accessibility as a Primary Leverage Point

Transportation emerged as the clearest access bottleneck. Transfer convenience was the lowest-rated indicator, whereas perceived need for increased service frequency and expanded routes was the highest-rated item. These findings suggest that transportation is not simply one domain among many; it may function as an upstream condition that shapes the practical usability of other services. In rural settings where essential services are concentrated in township centers or other local hubs, older adults’ practical access to those services may be influenced by transportation availability, route coverage, transfer burden, and the accessibility of stops and destinations. The focus-group findings further showed that transportation inconvenience involved not only limited service frequency but also route restructuring, repeated transfers, and uneven access to alternative transportation options. These contextual findings help explain how changes in service organization may increase the physical and procedural burden of essential trips, particularly for residents of outlying communities.

The contrast between relatively favorable access to public administrative facilities and lower access to essential healthcare facilities is also important. This pattern suggests that access barriers may be especially consequential when services are specialized, centralized, or less frequently available. For older adults, healthcare access is closely tied to functional maintenance, chronic disease management, and quality of life. Transportation barriers that make healthcare and other essential services difficult to reach may reduce practical service usability and thereby have implications for rural aging-in-place planning. Participant accounts also indicated that low-cost taxi and community-based transportation options were not uniformly available across outlying areas, highlighting the importance of geographic equity in rural mobility support. From an applied gerontological perspective, transportation should therefore be treated as part of the care and service infrastructure for rural older adults, not merely as a mobility or infrastructure issue.

### 4.3. Last-Mile Walkability as a Transportation Usability Condition

The regression findings further clarify how access barriers may compound one another. Better walking-environment convenience was associated with lower odds of reporting high inconvenience with bus service frequency and routes. This suggests that perceived transportation inconvenience may reflect not only the supply of bus services but also the practical conditions required to use those services. Even when routes or schedules are available, poor last-mile walkability may reduce the usability of transportation by making it difficult or unsafe for older adults to reach bus stops, transfer points, healthcare facilities, or routine destinations.

This finding is consistent with an applied gerontological view of transportation as an access system rather than a single service. For older adults, the trip does not begin when they board a bus; it begins when they leave home and navigate the pedestrian environment. Sidewalk quality, curb conditions, slope, lighting, distance to stops, and perceived safety may all influence whether transportation is usable in practice. Rural aging-in-place strategies should therefore avoid separating transportation planning from walkability improvements. Instead, transportation support and last-mile environmental modification should be considered together.

The focus-group findings provided concrete local context for this association by describing abrupt level changes, steep ramps, discontinuous walking surfaces, minimally equipped roadside bus stops, and long distances between stops and homes. These accounts indicate that last-mile usability depends on continuity across the entire route rather than on the presence of any single accessible feature.

### 4.4. Digital and Information Access as a Complementary Access Dimension

Digital access difficulty was not statistically significantly associated with high inconvenience with bus service frequency and routes in the adjusted model. Therefore, the present regression findings do not establish digital access difficulty as a predictor of perceived transportation inconvenience, and the study hypothesis concerning this relationship was not supported. Nevertheless, the descriptive findings indicated substantial difficulty with digital information and service access among respondents. This suggests that digital access remains relevant to the broader everyday access framework, although its relationship with transportation usability requires further investigation. Participant accounts complemented the descriptive findings by showing that digital and information barriers involved difficulty using kiosks and automated terminals, obtaining readable timetable information, and completing mobile-based reservation procedures. They also highlighted the importance of large-print information, telephone support, and human-assisted alternatives. These qualitative findings support treating digital and information access as a distinct dimension of service usability, while the regression results do not establish a direct association with transportation inconvenience.

Transportation and essential-service access are increasingly mediated by digital information, including schedules, route changes, booking systems, public service announcements, and healthcare-related procedures. Digital inclusion may therefore be considered a complementary component of rural aging-in-place support rather than an intervention directly supported by the regression findings. Such support may include parallel non-digital channels, telephone guidance, in-person assistance, simplified information formats, and local intermediaries who can help older adults navigate service systems.

### 4.5. Interpreting Limited Subgroup Differences in the ECBI

The Everyday Convenience Barrier Index did not differ significantly by sex or between the township center and outlying townships. These null findings should be interpreted cautiously and should not be taken as evidence that individual or geographic differences are absent. Rather, within this exploratory composite measure, no clear subgroup differences were detected. Because the ECBI combines several distinct access domains, potentially different patterns across individual domains may not be reflected in the overall composite score.

The absence of a significant center–outlying difference in the composite index may also reflect the nature of the exploratory composite. Differences in one domain may be offset by similarities or contrasting patterns in another. For example, township-center residents and outlying residents may experience different configurations of access barriers, but these differences may not necessarily appear as a large mean difference in the overall composite index. From a policy perspective, these findings suggest the value of considering both broad baseline improvements and locally targeted interventions. County-wide strategies may address shared access barriers, while more specific supports may be needed for villages or groups experiencing particular combinations of transportation, healthcare, digital, or environmental constraints.

### 4.6. Exploratory Use of the ECBI for Summarizing Multidomain Access Barriers

In this study, the ECBI provided an exploratory summary of multidomain everyday access barriers relevant to rural aging in place. By combining indicators of healthcare access, transportation inconvenience, walkability, digital access, and wayfinding, the index was used to consider multiple access barriers together rather than only as separate domains. This approach is relevant to applied gerontology because the combined accessibility of everyday environments and service systems may influence older adults’ daily functioning, autonomy, and ability to use essential services.

Importantly, the ECBI should not be interpreted as a validated psychometric scale or as a measure of a single latent construct. Its components represent conceptually distinct access barriers and were combined with equal weighting for exploratory purposes. The index should therefore be viewed as a descriptive composite that summarizes selected access barriers within the present study. Further research is needed to examine its construct validity, alternative weighting approaches, reproducibility, and associations with relevant aging-in-place outcomes before it can be considered for broader application.

Despite these limitations, the exploratory use of the ECBI illustrates one approach to examining multiple everyday access barriers simultaneously. Future studies using larger and more diverse samples should determine whether such a composite approach provides explanatory or predictive value beyond the analysis of individual access domains.

### 4.7. Operational Safety and Rural Feasibility

Night-time walking safety was another salient concern. A substantial proportion of respondents reported threats to night-time walking safety, and the most frequently preferred streetlight option was wavelength-adjusted lighting designed to minimize crop-growth impacts. This finding highlights the importance of rural feasibility in applied gerontological intervention design. Safety interventions in rural areas are shaped not only by pedestrian needs but also by agricultural practices, local budgets, energy use, and community acceptance. The focus-group findings clarified that these safety risks were partly operational: streetlights near farmland were sometimes switched off during growing seasons, leaving some residents to travel along dark village roads. This context helps explain the preference for wavelength-adjusted lighting and demonstrates why rural safety interventions must balance pedestrian safety with agricultural feasibility.

This result suggests that rural aging-in-place interventions should address operational conditions, not only physical infrastructure. Lighting schedules, lighting type, maintenance, and coordination with agricultural concerns may influence whether safety interventions are acceptable and sustainable. In rural communities where major infrastructure projects are difficult to implement quickly, operational adjustments may offer feasible ways to improve perceived safety and everyday mobility.

### 4.8. Implications for Applied Gerontological Practice and Policy

The findings have several implications for applied gerontological practice and policy in rural communities. Taken together, they support intervention priorities that address transportation, last-mile walkability, service navigation, digital inclusion, and locally embedded community care as interconnected components of rural aging-in-place support.

**Figure 2:**
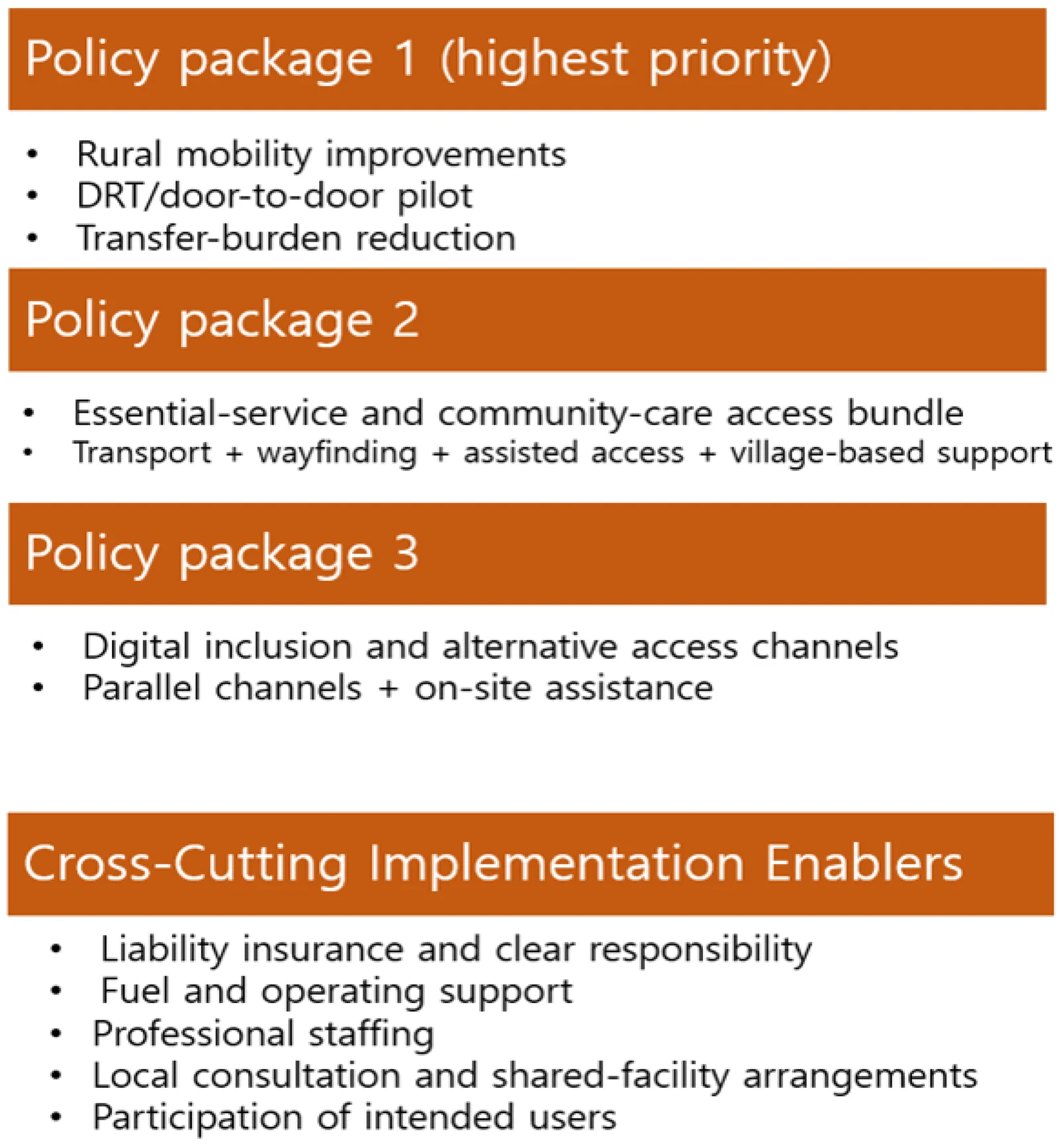
Applied gerontological intervention priorities for addressing compounded access barriers relevant to rural aging-in-place planning. The three policy packages represent prioritized intervention areas, whereas the cross-cutting implementation enablers apply across all packages. Abbreviation: DRT, demand-responsive transportation.

First, rural mobility interventions should focus on reducing transfer burden and improving the usability of transportation for essential trips. This may include expanding service frequency or routes where feasible, piloting demand-responsive transportation, strengthening door-to-door or semi-flexible options for high-need trips, and coordinating transportation with healthcare appointments and community care services. The focus-group findings further indicated that community-supported transportation requires practical implementation arrangements, including liability insurance, fuel and operating support, clear responsibility in the event of an accident, and predictable service schedules. Figure 2 summarizes the applied gerontological intervention priorities derived from these findings.

Second, transportation interventions should be paired with last-mile walkability improvements. Improvements around bus stops, healthcare facilities, community centers, and routine destinations may increase the practical value of existing transportation services. In this sense, walkability improvements are not merely environmental enhancements; they are part of the access infrastructure for rural aging in place.

Third, essential-service access should be supported through wayfinding and assisted navigation. The high need for barrier-free maps and signage suggests that older adults may benefit from clearer, more accessible information about service locations, routes, transfers, and available support. Wayfinding should be understood broadly to include physical signage, printed materials, telephone guidance, and in-person support. Such supports may be especially important for older adults who face both mobility constraints and digital access difficulty.

Fourth, digital inclusion should be integrated into rural aging-in-place strategies. The descriptive and focus-group findings suggest that digital difficulty may impede access to services that are increasingly mediated through online systems. Rural digital inclusion should therefore include practical assistance, alternative access channels, and service designs that do not assume digital independence among older residents.

These findings are consistent with prior qualitative evidence from rural South Korea, in which social welfare and care-service practitioners identified fragmented service delivery, limited accessibility, workforce burden, and the need for locally coordinated integrated care systems [28]. Community-based care strategies may be more effective when connected with transportation assistance, service navigation, digital support, and environmental improvements. Rather than treating community care as a separate service sector, rural aging-in-place planning should link care, mobility, information, and environmental usability into integrated intervention bundles.

### 4.9. Transferability Beyond South Korea

Although this study was conducted in one rural county in South Korea, its findings may be relevant to rural communities in East Asia and other rapidly aging regions where older adults remain in geographically dispersed settlements while healthcare, transportation, administrative, and commercial services become increasingly concentrated in local centers. The specific institutional context of South Korea is unique, but the underlying access problem is not. Many rural communities face population aging, youth outmigration, reduced service density, limited transportation options, digitalization of public services, and increasing pressure to support aging in place with constrained local resources.

The study therefore offers analytic transferability rather than statistical generalizability. The bottleneck-chain perspective may help researchers, local governments, and aging-service providers in other rural settings identify how everyday access barriers accumulate and where intervention priorities may be most effective. The exploratory ECBI used in this study provides a preliminary approach to summarizing selected multidomain barriers, but requires further testing before application in other populations.

### 4.10. Limitations and Future Research

This study has several limitations. First, the study was cross-sectional and conducted in a single rural county, which strictly precludes causal inference and limits statistical generalizability. Accordingly, the directional links illustrated in the conceptual model (Figure 1) represent hypothesized, interdependent bottleneck sequences derived from contextual synthesis rather than verified causal pathways. However, the purpose of the study was not to produce nationally representative estimates or test causal directions, but to examine how multidomain access barriers operate in a dispersed rural setting and to develop an analytically transferable framework for intervention prioritization.

Second, because this study was based on a secondary dataset originally collected for administrative planning, certain potentially critical socioeconomic covariates—such as household income, educational attainment, specific living arrangements (e.g., living alone), and driving status—were not available in the dataset. Although the logistic regression model controlled for key demographic characteristics (sex, age group), settlement structure, functional vulnerability (mobility-aid use while walking), and built-environmental conditions, the omission of unmeasured socioeconomic factors may have introduced residual confounding in predicting perceived transportation inconvenience. Future research utilizing primary cohort designs should incorporate comprehensive socioeconomic and detailed clinical variables to examine potential confounding or moderation effects more rigorously.

Third, the study did not directly measure intention to age in place, actual residential continuity, institutionalization, autonomy, or community participation. Accordingly, the findings should be interpreted as identifying everyday access conditions and intervention priorities relevant to aging-in-place planning rather than as demonstrating effects on aging-in-place outcomes.

Fourth, the qualitative inquiry relied on a single focus group comprising three township-level community welfare council members. While these participants served as highly experienced key informants with specialized, comprehensive knowledge of local service delivery systems, rural living realities, and vulnerable older populations, the small sample size inevitably constrains thematic data saturation and may not capture the full, heterogeneous spectrum of grassroots older residents’ lived experiences. Methodologically, this session functioned closer to an expert key-informant consultation aimed at contextualizing survey patterns rather than an exhaustive qualitative inquiry seeking broad demographic representation. Future research should conduct multi-stakeholder in-depth interviews and participatory co-design workshops engaging larger, diverse cohorts of older adults, informal family caregivers, transport providers, and municipal officials.

Fifth, the ECBI was an exploratory, equally weighted composite. It was useful for summarizing multidomain access bottlenecks, but it should not be interpreted as a validated measure. Its components were selected based on their conceptual relevance to everyday access within the available dataset. Future studies should examine alternative weighting approaches, construct validity, reproducibility, and associations with outcomes such as healthcare use, unmet service needs, perceived autonomy, social participation, functional maintenance, or intention and ability to age in place.

## 5. Conclusions

This study identified multidomain everyday access barriers in a dispersed rural county and suggested that practical service usability reflects interacting conditions involving transportation, healthcare access, last-mile walkability, digital and information access, wayfinding, and operational safety. By integrating survey findings with focus-group thematic findings, the study identified coordinated intervention priorities relevant to rural aging-in-place planning, including mobility improvements, walkability, assisted access, digital inclusion, and locally feasible implementation support. The exploratory ECBI provided a secondary summary of selected access barriers rather than a validated measure. Because aging-in-place outcomes were not directly assessed, the findings should be interpreted as identifying access conditions and intervention priorities relevant to aging-in-place planning rather than as demonstrating effects on residential continuity or other aging-in-place outcomes.

## Figures and Tables

**Table 1:** Participant Characteristics (N = 200).

Characteristic	Category	n (%)
Sex	Men	71 (35.5)
	Women	129 (64.5)
Age group	40s–50s	6 (3.0)
	60s	66 (33.0)
	70s	86 (43.0)
	80 years and older	42 (21.0)
Settlement structure	Township center	22 (11.0)
	Outlying townships	178 (89.0)
Mobility-aid use while walking	Yes	69 (34.5)
	No	131 (65.5)

Note. Percentages may not total 100 because of rounding. Outlying townships included eight myeon areas surrounding the township center. Overall, 194 participants (97.0%) were aged 60 years or older.

**Table 2:** Key Domain Indicators and Needs Related to Everyday Access and Aging in Place (0–100 Scale) (N = 200).

Domain	Indicator	Direction	Result
Physical accessibility	Access to/use of public administrative facilities	Higher = better	74.4
	Access to/use of essential healthcare facilities	Higher = better	29.4
Walking environment	Convenience of sidewalks and pedestrian routes	Higher = better	40.9
Transportation accessibility	Convenience of transfers when using local buses	Higher = better	16.6
	Need for increased service frequency and expanded routes	Higher = greater need	95.3
Digital access difficulty	Difficulty accessing digital information/services	Higher = greater difficulty	67.0
Safety and operations	Threats to night-time walking safety	% reporting yes	70.5
	Preferred streetlight option: wavelength-adjusted lighting	% selected	68.0
Community care	Support for village-based community care for aging in place	Higher = stronger support	85.4
Wayfinding and information	Need for barrier-free information (maps/signage)	Higher = greater need	79.0

Note. For Likert-type items, mean scores were transformed to a 0–100 scale using (x − 1)/4 × 100, where x represents the mean item score. Percentages represent the proportion endorsing or selecting the item.

**Table 3:** Subgroup Differences in the Everyday Convenience Barrier Index and Streetlight Operation Preference (N = 200).

Analysis	Group/Category	Result	Test Statistic	*p*	Effect Size
ECBI by sex	Men (n = 71)	M = 0.051, SD = 0.385	F(1, 198) = 2.039	0.155	eta squared = 0.010, 95% CI [0.000, 0.054]
	Women (n = 129)	M = −0.028, SD = 0.373			
ECBI by settlement	Township center (n = 22)	M = 0.041, SD = 0.373	t(198) = 0.539	0.590	Cohen’s d = 0.122, 95% CI [−0.321, 0.565]
	Outlying townships (n = 178)	M = −0.005, SD = 0.380			
Streetlight preference by sex	Wavelength-adjusted lighting	Men = 53; Women = 83; Total = 136 (68.0%)	chi-square(3) = 2.323	0.508	Cramer’s V = 0.108

Note. ECBI = Everyday Convenience Barrier Index. Higher ECBI values indicate greater barriers. Equal variances were assumed for the settlement comparison (Levene’s F = 0.096, *p* = 0.757). The chi-square assumption check indicated a minimum expected count of 5.32 and no cells with expected counts below 5.

**Table 4:** Logistic Regression Predicting High Inconvenience With Bus Service Frequency and Routes Among Adults Aged 60 Years and Older (N = 194).

Predictor	Category/Unit	aOR	95% CI	*p*
Sex	Women vs. men	0.938	[0.482, 1.824]	0.851
Settlement structure	Outlying townships vs. township center	1.115	[0.369, 3.373]	0.847
Mobility-aid use	Yes vs. no	1.218	[0.460, 3.224]	0.691
Walking-environment convenience	Per 1-point increase	0.541	[0.366, 0.800]	0.002
Digital access difficulty	Per 1-point increase	1.411	[0.997, 1.997]	0.052
Age group	70 s vs. 60 s	1.634	[0.749, 3.564]	0.218
	80 years and older vs. 60 s	0.687	[0.203, 2.325]	0.546

Note. Outcome = 1 if the respondent selected 1 or 2, corresponding to very inconvenient or inconvenient; otherwise, 0. Reference categories were men, 60s, township center, and no mobility-aid use. Model fit: omnibus chi-square(7) = 28.890, *p* < 0.001; Cox & Snell R squared = 0.138; Nagelkerke R squared = 0.188. aOR = adjusted odds ratio; CI = confidence interval.

**Table 5:** Joint Display Integrating Quantitative and Focus-Group Findings.

Quantitative Finding	Focus-Group Finding	Integrated Interpretation	Intervention Priority
Transfer convenience was very low (16.6), need for increased service frequency and expanded routes was high (95.3), and access to essential healthcare facilities was low (29.4).	Participants described reduced bus services, route restructuring, repeated transfers, uneven access to low-cost taxi services, and substantial burdens in completing essential trips.	Quantitative and qualitative findings converged in identifying transportation as the primary everyday access bottleneck. Transportation difficulty involved both limited service supply and the procedural and physical burden of rural travel.	Improve service frequency and route coverage; reduce transfer burden; develop demand-responsive, door-to-door, or community-supported transportation for essential trips.
Walking-environment convenience was relatively low (40.9). Better walking-environment convenience was associated with lower odds of high transportation inconvenience (aOR = 0.541, 95% CI [0.366, 0.800], *p* = 0.002).	Participants described steep and discontinuous routes, inaccessible ramps and trail surfaces, limited facilities at remote bus stops, and long distances between stops and homes.	The qualitative findings contextualized the regression result by showing how last-mile conditions may affect the practical usability of transportation beyond bus frequency and routes alone.	Pair transportation improvements with accessible pedestrian routes, bus-stop facilities, ramps, crossings, and connections to healthcare and community destinations.
Threats to night-time walking safety were reported by 70.5%, and wavelength-adjusted lighting was selected by 68.0% as the preferred streetlight option.	Participants described streetlights being switched off because of concerns about crop growth, leaving some residents to walk long distances on dark roads with risks from wild animals.	Safety barriers reflected both physical infrastructure and local operational conditions. Effective lighting strategies must account for pedestrian safety and agricultural feasibility.	Introduce context-sensitive lighting options, including wavelength-adjusted lighting, appropriate operating schedules, and maintenance strategies developed with local consultation.
Digital access difficulty was high (67.0), but it was not statistically significantly associated with transportation inconvenience in the adjusted model (aOR = 1.411, 95% CI [0.997, 1.997], *p* = 0.052).	Participants described difficulty using kiosks and automated systems, obtaining readable bus information, and completing mobile-based reservations without assistance.	The strands were complementary rather than fully convergent. Digital and information access represented an important independent service-access barrier, but the study did not establish it as a predictor of transportation inconvenience.	Provide large-print and clearly displayed information, telephone and in-person assistance, printed materials, and parallel non-digital service channels.
Support for village-based community care was high (85.4), and need for barrier-free wayfinding information was high (79.0).	Participants proposed using village halls and senior centers for locally embedded care and assistance, developing accessible information about facilities, and supporting community transportation through insurance, fuel, and operating arrangements.	The focus-group findings expanded the survey results by identifying the local infrastructure and administrative conditions needed to translate expressed needs into feasible interventions.	Link village-based care with mobility, digital assistance, wayfinding, and service navigation; provide institutional support for community-led initiatives.

## Data Availability

The data presented in this study may be available from the corresponding author upon reasonable request and subject to ethical and institutional approval. The data are not publicly available due to privacy and ethical restrictions.
